# Smaller intercondylar notch size and smaller ACL volume increase posterior cruciate ligament rupture risk

**DOI:** 10.1007/s00167-022-07049-5

**Published:** 2022-07-15

**Authors:** K. S. R. van Kuijk, M. Reijman, S. M. A. Bierma-Zeinstra, D. E. Meuffels

**Affiliations:** 1 Department of Orthopedic Surgery and Sports Medicine, Erasmus MC University Medical Center Rotterdam, Doctor Molewaterplein 40, 3015 GD Rotterdam, The Netherlands; 2 Department of General Practice, Erasmus MC University Medical Center, Rotterdam, The Netherlands; 3 Department of Radiology, Albert Schweitzer Hospital, Dordrecht, The Netherlands

**Keywords:** Posterior cruciate ligament, Anatomical risk factors, Knee, Ligament injury, Sports injury

## Abstract

**Purpose:**

Little is known about risk factors for sustaining a posterior cruciate ligament (PCL) rupture. Identifying risk factors is the first step in preventing a PCL rupture from occurring. The morphology of the knee in patients who ruptured their PCL may differ from that of control patients. The hypothesis was that the intercondylar notch dimensions, 3-D volumes of the intercondylar notch and, the 3-D volumes of both the ACL and the PCL were correlated to the presence of a PCL rupture.

**Methods:**

The magnetic resonance imaging (MRI) scans of 30 patients with a proven PCL rupture were compared to 30 matched control patients with proven intact ACL and PCL. Control patients were selected from patients with knee trauma during sports but without cruciate ligament injury. Patients have been matched for age, height, weight, BMI, and sex. The volumes of the intercondylar notch and both the ACL and PCL were measured on 3D reconstructions. Second, the bicondylar width, the notch width, and the notch width index were measured of all subjects. The relationship between our measurements and the presence of a PCL rupture was analysed.

**Results:**

The results show a significant difference in the volumes of the intercondylar notch and the ACL between patients with a ruptured PCL and control patients. Patients with a PCL rupture have smaller intercondylar notch volumes and smaller ACL volumes. There were no significant differences in the bicondylar width, notch width, and notch width index. In the control patients, a significant correlation between the volume of the PCL and the volume of the ACL was found (0.673, *p* < 0.001).

**Conclusion:**

Patients with a PCL rupture have smaller intercondylar volumes and smaller ACL volumes when compared to control patients. Second, patients with smaller ACL volumes have smaller PCL volumes. This study shows, for the first time, that there are significant size and volume differences in the shape of the knee between patients with a PCL rupture and control patients.

**Level of evidence:**

IV.

**Supplementary Information:**

The online version contains supplementary material available at 10.1007/s00167-022-07049-5.

## Introduction

Injuries to the posterior cruciate ligament (PCL) occur in approximately 1–4% of all sport-related traumatic knee injuries, depending on the type of sport [14, 17]. With the growing number of athletes and especially of competitive athletes [10], the absolute number of PCL injuries is growing and is likely to keep on growing in the next couple of years [3, 11].

In the short term, a PCL rupture, in most cases, causes pain and posterior laxity and reduces an individual’s ability to take part in sports. In the long term, deficiency of the PCL results in abnormal kinematics and increased contact pressures in the medial and patellofemoral compartments of the knee and may increase strain on the posterolateral knee structures, placing them at risk of subsequent injury [9, 25]. Long-term studies have found that degenerative changes after PCL injury occur primarily in the medial and patellofemoral compartments [2, 6, 8, 17, 20]. PCL injuries are still often overlooked during primary care [18, 23], resulting in delayed diagnosis and delayed treatment. Therefore, risk factors need to be identified. Recently, van Kuijk et al. found that the size and shape of the intercondylar notch and the tibial eminence are related to the risk of sustaining a PCL rupture [13]. This study is conducted on plain radiographs. The knee, however, is a 3-Dimensional, complex joint, best analysed on MRI. However, until this date, no studies using MRI have been conducted to investigate the morphological features of the PCL deficient knee. It might be possible that the shape of the intercondylar notch has a positive correlation with the soft tissue moving through it, such as the PCL.

Therefore, the purpose of this study was to investigate if the volumes of the cruciate ligaments, the sizes of the intercondylar notch, and the intercondylar notch dimension are related to the risk of sustaining a PCL rupture.

## Material and methods

The institutional ethics review board of the Erasmus MC, Rotterdam, approved this study (MEC-2017-422).

### Patient selection

Patients with a PCL rupture and control patients were selected from patients visiting our outpatient clinic of the Erasmus Medical Center, Rotterdam, The Netherlands, between January 2003 and May 2014. Patients and controls had to be practising pivoting sports competitively during the time of injury. Patients were only included if they had an isolated PCL rupture, confirmed by MRI or arthroscopy. Controls were selected from patients who had sustained a meniscal tear or a combined medial collateral ligament and meniscus injury and had an intact PCL and intact ACL confirmed by MRI or arthroscopy. Patients were excluded if they had radiographic evidence of knee osteoarthritis Kellgren and Lawrence score two or higher because secondary bone formation could change the notch shape and decrease or alter the volume. Patients were matched for age, height, weight, BMI, and sex.

The institutional ethics review board approved this study.

### MRI protocol and segmentation

MR images were obtained using MRI scanners with a magnetic field strength of 1.0, 1.5, or 3.0 Tesla.

The MRI sequences used are sag PD, sag T2 FS, Cor PD FS, Coronal T2 TSE, axial T2 FS, axial PD. Patients' legs were positioned neutrally. To assess PCL injury, and the measurements in the knee described below, we used sagittal and coronal proton density-weighted turbo spin-echo (TSE) sequences (slice thickness 1 mm, repetition time (TR)/echo time (TE), 2700/27 ms), and the coronal T2-weighted TSE sequence with fat saturation (slice thickness 1 mm, TR/TE 5030/71 ms).

### ACL and PCL volume measurements

**Fig. 1:**
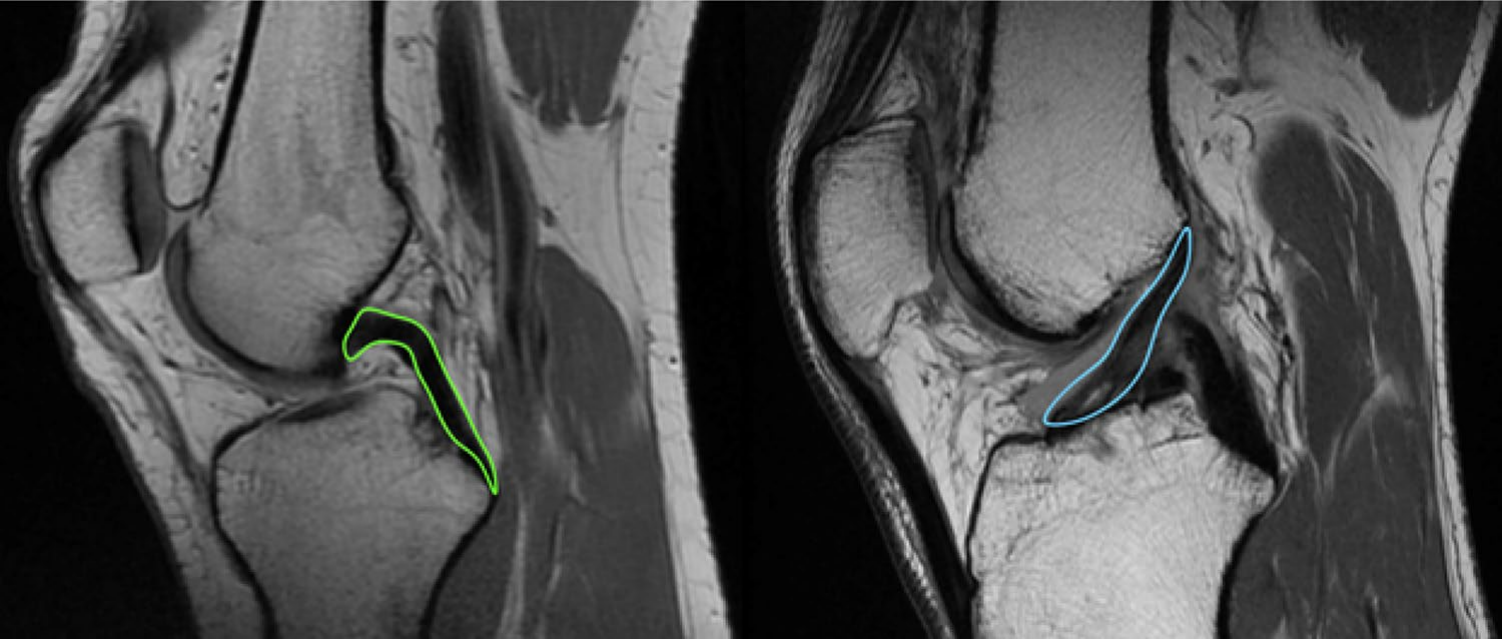
Sagittal view MRI of the knee, in which the PCL (left, green) and the ACL (right, blue) are outlined

The volumes of the ACL in both groups, and the PCL in the controls, were obtained from sagittal T1-weighted series. Osirix software (open-source medical imaging software for MacOS X [Apple, Cupertino, CA]; OsiriX, Geneva, Switzerland) was used to manually outline the ACL and PCL. This method was tested and found to be reliable and accurate in previously conducted research[28]. The software calculated the volume as the sum of surface multiplied by the slice thickness (1 mm), the volumes were presented in two decimals. (see Fig. 1). Furthermore, the correlation between the size of the ACL and the PCL in control patients was analysed.

### Femoral notch measurements

**Fig. 2:**
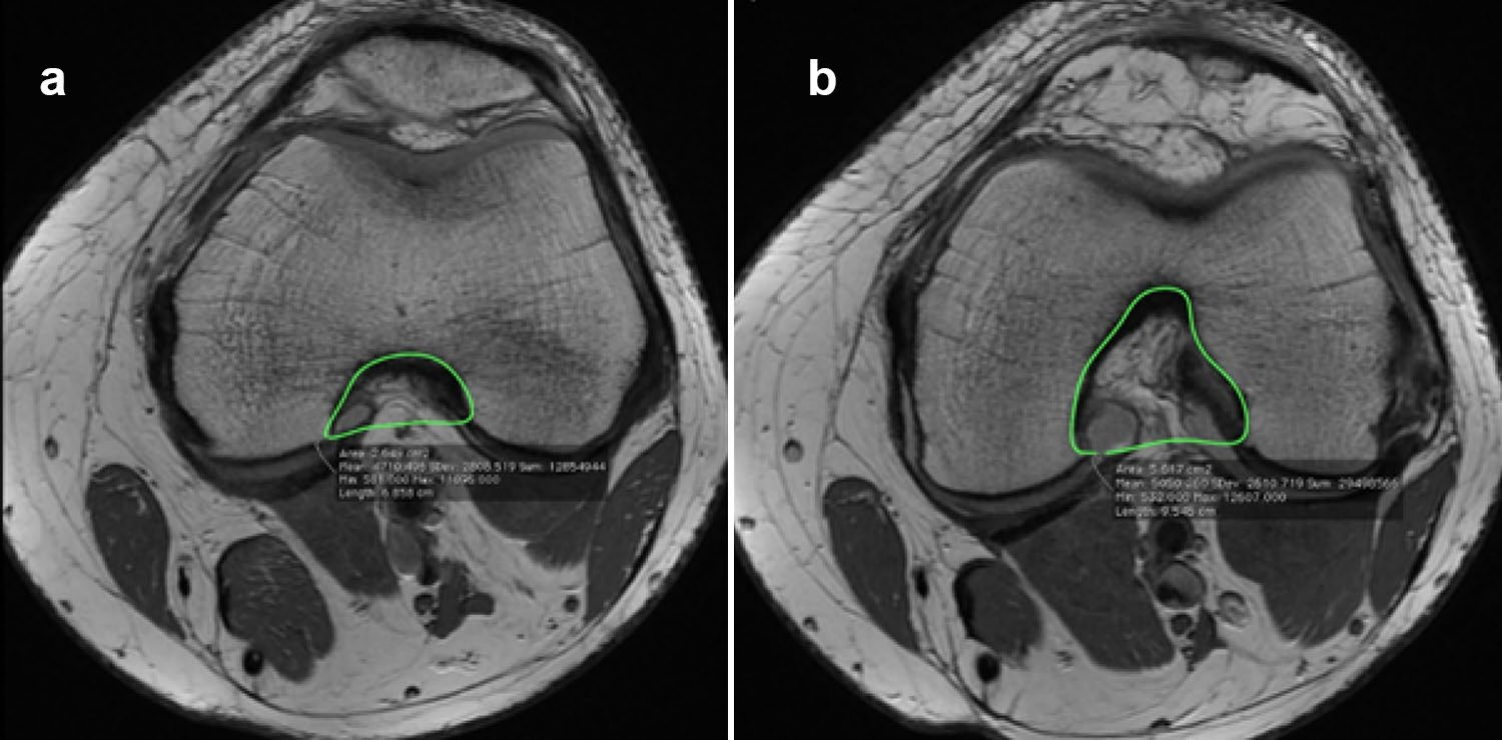
Axial MRI image of the knee, **A** most proximal, **B** most distal of the intercondylar notch

The boundaries of the intercondylar notch were measured according to Van Eck et al., who previously described the boundaries of the intercondylar notch [4]. The proximal border of the notch is defined as the image in which both femoral condyles were first clearly visible (Fig. 2A). The distal border of the notch is defined as the last image in which the condyles were continuous (Fig. 2B).

**Fig. 3:**
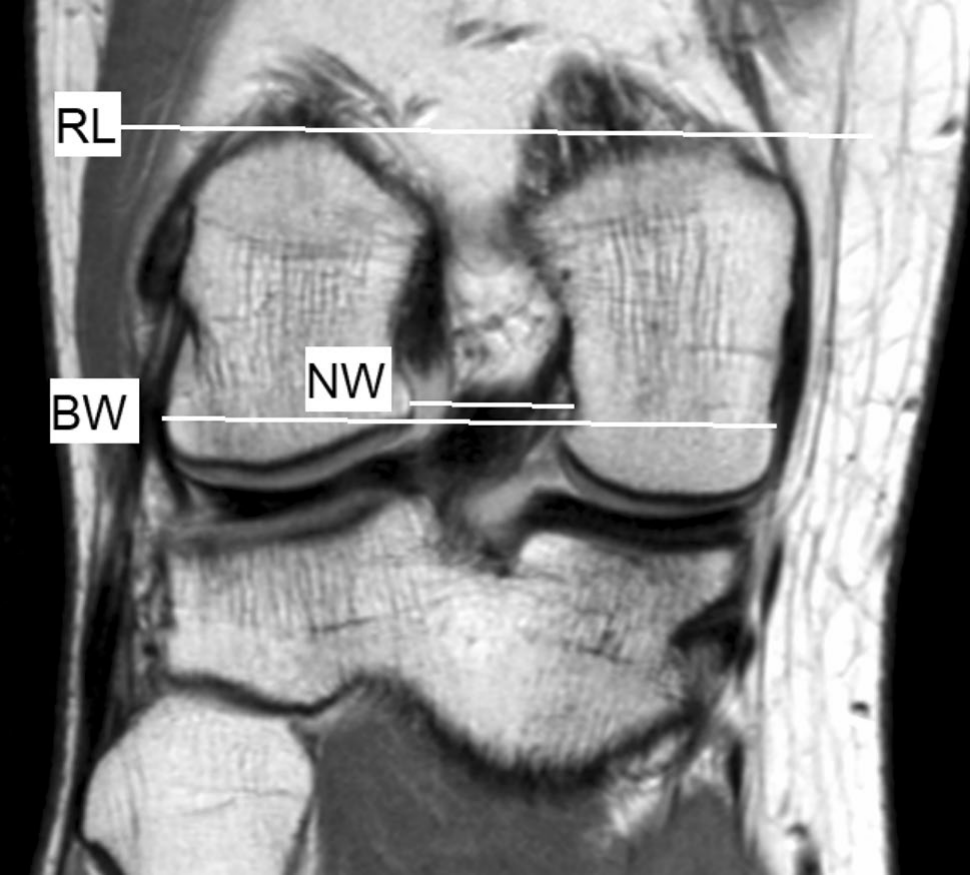
Coronal MRI of the knee, with the described measurements. *RL* reference line, *NW* notch width, *BW* bicondylar width

Additionally, the notch width index (NWI) was measured according to the method first described by Staeubli et al. [21] and later modified by Whitney et al. [26]. A reference line (RL) is defined as a tangent to the posterior subchondral aspect of both femoral condyles. All femoral widths are measured parallel to this reference line. In the coronal plane, the following measurements were applied: bicondylar width (BW) and notch width outlet (NW). The notch width index was calculated by dividing the NW by the BW (Fig. 3).

A T2 coronal plane was used in each patient to measure BW and NW. The slice chosen in every knee was the plane in which the ACL and PCL cross one another as close as possible to the midsubstance of the ACL [26]. This point was typically found on the first slice anterior to the appearance of the roof of the intercondylar notch.

## Statistical analysis

The independent-sample t test was used to assess whether or not the volume of the intercondylar notch and the volumes of the ACL and PCL and the bicondylar width, the notch width, and the notch width index were significantly related to the presence of a PCL rupture. The results were not corrected for height, weight, BMI, age, and sex, since we matched our cases with our patients on those factors. A value of 0.05 was chosen as the level of significance.

The reliability of the measurements performed in this study was established using interclass correlation coefficients (ICCs). Twenty-eight MRI scans were randomly selected to be assessed a second time, two weeks after initial measurements. The observer did not know the values of previous measurements. A post hoc power analysis was performed, showing that the current study with 30 patients included in each group, had a power of 94.9%. The calculation showed that 28 patients in the control group and 28 patients with a PCL rupture were needed to reach a sufficient power. With 30 patients, the power was considered excellent.

The study population consisted of two groups of 30 patients matched for age, height, weight, BMI, and sex. There were six females (20%) in each group. The demographic data for each group are shown in Table 1.

**Table 1:** Characteristics of the patients

	PCL injured (*n* = 30)	PCL intact (*n* = 30)
Age (years)	40 ± 13	38 ± 12.0
Length (cm)	180 ± 11	179 ± 9
Weight (kg)	80.3 ± 12	82.5 ± 12
BMI	24.3 ± 2.1	25.6 ± 3.0
Female, *n* (%)	6 (20%)	6 (20%)
Mean time between trauma and radiograph, mths (SD)	12.6 ± 16	6.9 ± 10

Data presented as mean with ± SD

*BMI* body mass index

## Results

### Measurements

Table 2 shows the means for the BW, NW, NWI, the volumes of the PCL and ACL, and the intercondylar sizes. A significant difference in the sizes of the intercondylar notch (*p* = 0.001) between patients with a PCL rupture and the control patients was found. Secondly, a significant difference between the volumes of the ACL (*p* = 0.039) in patients with a PCL rupture and patients with intact PCLs was found.

**Table 2:** Overview of measurements of the Bicondylar Width (BW), the Notch Width (NW), the Notch Width Index (NWI), the intercondylar volumes and the cruciate ligament volumes

(Mean ± SD)	PCL injured (*n* = 30)	PCL intact (*n* = 30)	*p* value
BW (mm)	7.8 ± 0.7	8.1 ± 0.5	(n.s.)
NW (mm)	2.1 ± 0.4	2.2 ± 0.3	(n.s.)
NWI	0.3 ± 0.1	0.3 ± 0.1	(n.s.)
Intercondylar volumes (cm^3^)	7.0 ± 2.2	8.6 ± 1.2	**0.001**
PCL volumes (cm^3^)	–	2.2 ± 0.4	
ACL volumes (cm^3^)	1.1 ± 0.3	1.3 ± 0.3	**0.039**

Measurements presented in mean with ± SD. Bold represent a *p* values < 0.05 are considered significant

Patients with a PCL rupture had smaller intercondylar sizes (7.0 cm^3^ ± 2.2 compared to 8.6 cm^3^ ± 1.2) and smaller volumes of the ACL (1.1 cm^3^ ± 0.3 compared to 1.3 cm^3^ ± 0.3), when compared to patients with an intact PCL.

There were no significant differences in the bicondylar width, notch width, and notch width index.

The correlation between the size of the PCL and the ACL in the control patients was found to be a positive correlation of 0.7. Meaning patients with smaller volumes of the PCL are more likely to have smaller volumes of the ACL.

When looking at sex, the intercondylar sizes (*p* = 0.02) and the volumes of the ACL in males were significantly larger than in the controls (*p* = 0.01). For the females, only the intercondylar volumes were significantly different between patients and controls (*p* = 0.001) (Table 3).

**Table 3:** Descriptives of measurements divided between males and females and between controls and injured patients

(Mean ± SD)	Males			Females		
	PCL injured (*n* = 24)	PCL intact (*n* = 24)	*p* value	PCL injured (*n* = 6)	PCL intact (*n* = 6)	*p* value
BW (mm)	79.8 ± 5.7	82.3 ± 3.6	(n.s.)	71.3 ± 5.4	73.9 ± 4.1	(n.s.)
NW (mm)	20.8 ± 3.4	22.1 ± 2.4	(n.s.)	21.2 ± 4.5	21.3 ± 3.0	(n.s.)
NWI	26.0 ± 3.9	26.9 ± 2.8	(n.s.)	29.5 ± 4.7	28.8 ± 3.7	(n.s.)
Intercondylar volumes (cm^3^)	7.5 ± 2.2	8.8 ± 1.1	**0.015**	5.0 ± 0.9	8.0 ± 1.3	**0.001**
PCL volumes (cm^3^)	–	2.3 ± 0.4	–	–	1.7 ± 0.4	–
ACL volumes, controls only (cm^3^)	1.2 ± 0.3	1.4 ± 0.3	**0.013**	1.0 ± 0.2	1.0 ± 0.3	(n.s.)

Measurements presented in mean with ± SD. Bold represent a *p* values < 0.05 are considered significant

The ICCs were considered excellent with values of 0.906 for BW (95% CI 0.807–0.955), 0.993 for NW (95% CI 0.984–0.997) and 0.952 for NWI (95% CI 0.899–0.977).

## Discussion

The most important findings of this study are that patients with a PCL rupture had smaller intercondylar sizes and had smaller ACL volumes. To our knowledge, this is the first study to find anatomical risk factors for sustaining a PCL rupture. In a recent study on plain radiographs of the knee, van Kuijk et al. found that the shape of the intercondylar notch plays an important role in the PCL deficient knee [13]. With this current study, using MRI reconstructions of the intercondylar notch and the volumes of the PCL and ACL, it becomes clearer what specific anatomical features contribute to these findings on 2D plain radiographs.

Possibly, patients with smaller ACL volumes are also at greater risk for sustaining a PCL rupture. The absolute mean difference was 17 cubic millimetres, making it a statistically significant difference. Although the absolute difference is relatively small, raising the question if this would be clinically important, it is a difference of more than 10% in volume. Our opinion is, that more than 10% difference in size would be clinically relevant, and as stated, it was statistically different too. A positive correlation between the volumes of the PCL and the ACL was found, and although the sample size is small, it can be assumed that patients with smaller ACL volumes had smaller PCL volumes. Smaller PCLs can withhold less force and are more prone to rupture [15, 24]. Furthermore, the size of the PCL (and of the ACL) is correlated with the size of the intercondylar notch [16, 22]. It is well accepted that the volumes of the intercondylar notch, the PCL, and the ACL are all positively correlated. A question still unresolved is whether patients with smaller cruciate ligaments and smaller intercondylar notches rupture their ligament because of the relatively smaller force they can withhold, or because of chronic or acute damage because of notch impingement to the cruciate ligaments [1, 5, 19, 27]. However, notch impingement may play a lesser role in the risk for sustaining a PCL rupture, because of the anatomical position of the PCL posterior in the knee.

When comparing males to females, we find a similar result, with the note that we only have a small group of females, thus the statistical power of these results is low.

It is difficult to compare the results of this study to previously conducted research since this study is the first to have investigated the volumes of the intercondylar notch and the volumes of the PCL and ACL in correlation with a PCL rupture. Research is abundant into the intercondylar notch and the ACL deficient knee (see Supplemental File) [12, 24, 26, 28]. These studies found similar results in the ACL deficient knee as we have found in the PCL deficient knee. This current study, therefore, adds important new information, proving the importance of the volumes of the cruciate ligaments and the sizes of the intercondylar notch.

The risk factors in the current study are possibly modifiable (for example, during surgery with notch plasty), it is uncertain if changing the notch dimension, can reduce the risk for future PCL rupture, and notch plasty results are debated in the literature.

A limitation is the low number of patients included in the study. Ideally, more patient would have been included, to have more power, although the study has an adequate power. However, few patients have a sports-related isolated PCL rupture. Because the consequences of this type of injury are severe, we are confident that research into the PCL rupture is necessary.

Degenerative changes of the knee could result in osteophyte formation and narrowing of the intercondylar notch. These changes happen more often after PCL rupture. This could mean that the results found were based on post-traumatic degenerative changes. Therefore, patients with Kellgren and Lawrence grade 2 or more were excluded, reducing this possible bias as much as possible.

While prevention programs are implemented for preventing an ACL rupture during sports [7], it has not been investigated if these programs or comparable programs can help to reduce the incidence of PCL ruptures. However, for preventing a disease or injury in general, risk factors must first be identified, and that is, in our opinion, the clinical relevance of our study. We are convinced, this study is an important first step in identifying risk factors, and we hope that we have inspired researchers to further investigate the results found. This study shows, for the first time, that there are significant differences in the shape of the knee between patients with a PCL rupture and control patients.

## Conclusion

This study shows that patients with a smaller intercondylar size and a smaller volume of the ACL are more prone to sustain PCL rupture. This is an important first step in identifying patients at risk for a PCL rupture. Identifying patients at risk is useful information if in the future, the risk of individuals for sustaining a PCL rupture might be reduced.

## Supplementary Information

Below is the link to the electronic supplementary material.

Supplementary file1 (DOCX 101 KB)
