# Supplementary material for: Smaller intercondylar notch size and smaller ACL volume increase posterior cruciate ligament rupture risk
Source: Knee Surg Sports Traumatol Arthrosc. 2022 Jul 15;31(2):449–54. doi: 10.1007/s00167-022-07049-5 (PMC9898422; doi:10.1007/s00167-022-07049-5)
Supplement: Supplementary file 1 — Supplementary file1 (DOCX 101 KB) [file 167_2022_7049_MOESM1_ESM.docx]

**Smaller intercondylar notch and cruciate ligament volume increase anterior cruciate ligament rupture risk.**

**Abstract**

Purpose: Anatomical variations of the knee have often been a subject of interest in finding predictors for an Anterior Cruciate Ligament (ALC) rupture. Due to differences in methodology, results vary between studies and therefore consensus is lacking. A recent study suggested that smaller intercondylar notches are related with an ACL rupture on 2D x-rays.

Our objective is to verify that intercondylar notch dimensions as well as 3-D volumes of the intercondylar notch and the 3-D volumes of both the ACL and the posterior cruciate ligament (PCL) are correlated to the risk of sustaining an ACL rupture.

Methods: We retrospectively compared Magnetic resonance imaging (MRI) scans of 121 patients with a proven ACL rupture to 92 control patients with proven intact ACL’s and PCL’s. Patients were selected for age, weight, height and sex (by manual selection). We measured the volumes of the intercondylar notch and ACL and PCL, the bicondylar width (BW), the notch width (NW), and the notch width index (NWI). Secondary, we compared the result between male and females.

Results: Patients with an ACL rupture had, on average, a smaller NW (P<.001), a smaller NWI (P<.001), smaller intercondylar volumes (P<.001) and smaller volumes of the PCL (P<.001). Secondary results showed that females have on average a smaller NW (P<.001), smaller volumes of the intercondylar notch (P<.001), ACL (P=.004) and PCL (P<.001). However, the NWI was not significantly different between the sexes (P=0.508).

Conclusion: A smaller notch dimension, smaller volumes of the intercondylar notch and smaller volumes of the PCL’s are related to the presence of an ACL rupture. Secondary, females have smaller volumes of the intercondylar notch, ACL’s and PCL’s, but do not have a smaller NWI, when compared to males.

Keywords: Anterior Cruciate Ligament ; femoral intercondylar notch; knee anatomy; risk factors;

**Introduction**

Does size matter? Recent studies suggested that the width of the intercondylar notch and the width of the tibia eminence are related to the risk of sustaining anterior cruciate ligament (ACL) rupture [[2](#_ENREF_2), [6](#_ENREF_6), [8](#_ENREF_8), [9](#_ENREF_9), [12](#_ENREF_12), [14](#_ENREF_14), [16](#_ENREF_16), [17](#_ENREF_17), [25](#_ENREF_25), [38](#_ENREF_38)] and a study showed that they have worse outcome after suffering an ACL injury [[10](#_ENREF_10)]. A hypothesis generating program, to objectively analyse the shape of the knee showed that the size and the width of the intercondylar notch is related to higher risk for sustaining an ACL rupture[[34](#_ENREF_34)] . However, some of these studies were performed on 2D radiographs, while the knee is a complex 3-dimensional structure. MRI imaging has the advantage over x-rays that not only the osseous structures but also the ligaments and other soft tissues can be assessed. Also, MRI has become general practice after a knee trauma, with an increasing number of MRI’s made each year [[1](#_ENREF_1)], providing a more widespread availability of MRI both in control patients, as in patients with an ACL rupture. These MRI’s of subjects, can be used for research into anatomical risk factors for ACL rupture.

Previously published research into the 3-Dimensional volumes of the intercondylar notch and the volumes of the cruciate ligaments looked at gender differences [[7](#_ENREF_7)]; other studies had smaller sample groups [[22](#_ENREF_22), [26](#_ENREF_26), [32](#_ENREF_32)]. Sturnick et al published a study in 2015 investigating the anatomical risk factors, but produced very small odds ratios [[30](#_ENREF_30)]. Uncontradictable evidence that a smaller intercondylar notch volume relates to a greater risk for sustaining an ACL rupture is still lacking.

In order to prevent ACL ruptures in athletes, we have to be able to identify a person who is at greater risk for sustaining an ACL rupture. Studies investigating predictors for an ACL rupture have, to date, failed to produce a usable predictor for an ACL rupture[[23](#_ENREF_23)]. Some predictors that are found in these studies are not modifiable, such as sex and genetic factors [[3](#_ENREF_3), [13](#_ENREF_13), [18](#_ENREF_18), [20](#_ENREF_20), [21](#_ENREF_21), [28](#_ENREF_28)]. When risk factors are determined, individuals can be counselled for example, to avoid pivoting sports, join neuromuscular prevention programmes or maybe have intercondylar notch surgery after an ACL ligament rupture, to prevent future re-rupture.

Therefore, the aim of our study was to provide evidence on 3-D MRI reconstructions that the volume and dimensions of the intercondylar notch and of the cruciate ligaments are determinants for sustaining an ACL rupture.

**Patients and Methods**

***Patients***

Patients included in this study were identified from a previously conducted prospective study at our Department of Orthopaedics; the KNee osteoArthritis anterior cruciate Ligament Lesion (KNALL)[[10](#_ENREF_10)] .The KNALL is a prospective observational study of 154 patients with an ACL rupture, who were treated either operatively or non-operatively. MRI’s were performed within 3 months after knee trauma. Patients were included in the KNALL study from January 2009 to November 2010, with a follow-up period of two years. All patients had suffered complete ACL rupture. For convenience, the term rupture is used in this paper for all patients with a complete ACL rupture.

***Control patients***

The control group consisted of 92 patients who had had a knee injury, either comprising of 1) meniscus injuries or 2) collateral ligament and meniscus injury. We performed the search in the database of our university medical centre with a time interval from January 2003 until February 2014. Inclusion criteria were: intact ACL and PCL, diagnosis proven by MRI or by arthroscopic surgery of the knee; MRI scans and knee X-rays of the knee were available; patients had to be practicing (pivoting) sports, on a regular basis, before the trauma. Table 1 shows the type of sports of the controls patients. Exclusion criteria where: radiographic evidence of knee osteoarthritis defined as Kellgren and Lawrence score 2 or higher.

Our study and the KNALL study where both approved by our hospitals ethical review commission.

***MRI protocol and Segmentation***

At baseline, MR images were obtained using MRI scanners with a magnetic field strength of 1.0, 1.5, or 3.0 Tesla. Patients' legs were positioned neutrally. All MRI examinations included a set of routine clinical MRI pulse sequences. To assess ACL features, we used sagittal and coronal proton density weighted turbo spin echo (TSE) sequences (slice thickness 1 mm, repetition time (TR)/echo time (TE), 2700/27 ms) and the coronal T2-weighted TSE sequence with fat saturation (slice thickness 1 mm, TR/TE 5030/71 ms). Differences in field strength are proven to be irrelevant diagnosing an ACL rupture [[5](#_ENREF_5), [19](#_ENREF_19)].

***ACL and PCL volume measurements***

The volumes of the PCL in both groups, and the ACL in the controls, were obtained from sagittal T1-weighted series. Osirix software (open-source medical imaging software for MacOS X [Apple, Cupertino, CA]; OsiriX, Geneva, Switzerland) was used to manually segment the ACL and PCL. This method was tested and found to be precise and accurate[[37](#_ENREF_37)]. The volume was calculated as the sum of the outlined areas multiplied by the slice thickness. (See figure 1).

We tested the correlation between the intercondylar notch volume and the volume of the PCL.

***Femoral notch measurements***

The volumes of the intercondylar notch were measured using Osirix software. Charlton et al[[7](#_ENREF_7)] and Van Eck et al[[33](#_ENREF_33)] previously described the boundaries of the intercondylar notch. The proximal border of the notch is defined as the image in which both femoral condyles were first clearly visible (Figure 2A). The distal border of the notch is defined as the last image in which the condyles were continuous (Figure 2B). We also measured the notch width index (NWI) according to a method first described by Staubli et al [[27](#_ENREF_27)] and later modified by Whitney et al [[35](#_ENREF_35)]. A reference line (RL) is defined as a tangent to the posterior subchondral aspect of both femoral condyles. All femoral widths are measured parallel to this reference line. In the coronal plane, the following measurements were applied: Bicondylar width (BW) and notch width outlet (NW). The Notch Width Index was calculated by dividing the NW by the BW. (figure 3).

A T2 coronal plane was used in each patient to measure BW and NW. The slice chosen in every knee was the plane in which the ACL and PCL cross one another as close as possible to the midsubstance of the ACL in the control cases. This point was typically found in the ACL injured group on the first slice anterior to the appearance of the roof of the intercondylar notch.

**Statistical analysis**

We used independent t-test to assess whether or not the volume of the intercondylar notch and the NWI were significantly related to the presence of an ACL rupture. We used the presence or absence of an ACL rupture as dependent factor and the volume of the intercondylar notch and the NWI as independent variables. We adjusted the analysis for BW, NW and NWI. We did not correct for weight, height, age and sex, since we selected our cases with our patients on those factors. We chose our controls slightly older age then our patients with an ACL rupture to make sure the control group had been longer at risk for sustaining an ACL during their longer period of practicing sports. A value of P < .05 was chosen as the level of significance.

Reliability of the measurements performed in this study was established using variance component analysis to estimate the variability within examiners. Intraclass correlation coefficients (ICCs) were then calculated for assessing intra-observer reliability. We randomly selected 28 MRI scans ( 14 of ACL ruptured patients and 14 of control patients) to be assessed a second time, two weeks after initial measurements. The observer did not know the values of previous measurements. The ICC were considered excellent with values of 0.9 for BW, 0.99 for NW and 0.95 for NWI and 0,92 for the PCL volume.

**Results**

The study population consisted of 121 patients who met our inclusion criteria with an complete ACL rupture and 92 controls (see flow chart). The mean age for the ACL injured was 31 (± 7.4) years versus 38 (±12.0) for the controls (p<0.05). The groups had comparable BMI, with mean BMI of 24.5 (± 3.9) for the ACL injured versus 25.6 (± 3.9) for the controls (p=0.1). The groups consisted of 37 females in the ACL injured group (30%) and 22 females in the control group (24%) (p=0.3). Both groups consisted of comparable numbers of males and females. As stated before, the control group were on average seven years older than our ACL ruptured group.

The correlation between the intercondylar notch volume and the volume of the PCL was a Pearson correlation of 0,44; (p < 0.001). There was a positive correlation between the volumes of the ACL and PCL’s of our control patients (Pearson correlation of 0,40; p < 0.001). All patients had an MRI of the affected knee within 2 months of the knee trauma.

154 patients from the KNALL study were assessed for eligibility

121 patients compared to 92 comparable control patients.

Database search

92 patients with an intact ACL and PCL, proven by MRI or arthroscopy.

Complete data.

121 with complete data and MRI from the KNALL study

33 patients not eligible

Incomplete data of missing MRI

**Measurements**

Table 2 shows the mean for the BW, NW, NWI and the Intercondylar volumes. The NW, NWI and the intercondylar volume were significantly different between patients with an ACL rupture and the control group, all p<.001. There was no significant difference between both groups in BW. We analyzed notch and ligament volumes by sex overall and then stratified according to case-control groups. (table 3 and table 4). Females had smaller intercondylar volumes than men (5.78 cm^3^ ± 1.74 compared to 7.03 cm^3^ ± 1.97; p<.001), and smaller volumes of the ACL and PCL. When divided in ACL injured and ACL intact groups, we found that for males the NW, NWI, intercondylar volumes and the volumes of the PCL were all significantly different. For the females, only the notch width and the intercondylar volumes were significantly different between the two groups.

**Discussion**

Our results showed that the volume of the intercondylar notch, the volume of the PCL, the Notch Width and the Notch Width Index are significant related to the presence of an ACL rupture. These findings provide evidence that a smaller volume of the intercondylar notch is related to the risk of sustaining an ACL rupture, as is the notch width and the notch width index. This study provides further evidence that the intercondylar notch width and the intercondylar notch volume play an important role in ACL injuries.

A smaller volume of the PCL is also significantly related to the presence of an ACL rupture. The most accepted explanation for this is that a smaller intercondylar notch holds a smaller PCL. Our study found an positive correlation between the intercondylar notch volume and the volume of the PCL (Pearson correlation of 0,44; p > 0.001). Although we were unable to measure the volumes of the ruptured ACL’s, there was a positive correlation between the volumes of the ACL and PCL’s of our control patients (Pearson correlation of 0,40; p > 0.001), suggesting that patients with an ACL rupture had smaller ACL’s.

Previous studies focussing on the morphology of the knee had conflicting results [[7](#_ENREF_7), [37](#_ENREF_37)]. This could be due to differences in methods and smaller group sizes where we used a larger group of patients and controls. There are few studies focussing on the volume of the intercondylar notch, while previous studies showed the importance of the shape of the intercondylar notch in relation with the ACL[[9](#_ENREF_9), [27](#_ENREF_27)].

The findings of our current studies confirm the hypothesis from our previously conducted study[[34](#_ENREF_34)] ; in this study we used a hypothesis free program to analyse shape variants between patients with an patients without an ACL rupture. The hypothesis generated with this 2D study was that a narrower intercondylar notch was related with an ACL rupture, as shown in this current study with 3D measurements.

The influence of intercondylar notch width as a risk factor for ACL tears is believed to be related to the size of the ACL[[24](#_ENREF_24)]. In a cadaver study by Stijak et al.[[29](#_ENREF_29)] in a large sample of 50 cadaver adult knees, notch width correlated significantly with ACL size in males.

Secondary results show that there is a statistical significant difference in intercondylar volume, PCL and ACL volume, notch width and bicondylar width between males and females, but that there is no significant difference in the notch width index between males and females. This means that the relative sizes of the intercondylar notch are comparable between males and females. The differences in BW, NW and volume of the intercondylar notch between males and females could be related to the fact that males are, in general, of greater size than females and thus the intercondylar notch of males holds larger ligaments. This could also account for the known fact that women have a threefold higher risk in sustaining an ACL rupture [[15](#_ENREF_15), [25](#_ENREF_25), [31](#_ENREF_31)]. A smaller notch, holds a smaller ACL volume[[12](#_ENREF_12), [26](#_ENREF_26)], which in turn can withhold less force[[36](#_ENREF_36)].

One of the strengths of our study is the use of a large group of patients with an ACL rupture and a large control group. They were selected from a database but had a similar exposure environment for suffering an ACL rupture, and even longer exposure time for obtaining an ACL rupture. We specifically included only controls practicing sports and with a knee trauma. This makes the two selected groups of patients more comparable than when we would have selected controls without a traumatic event.

A limitation of this study is that although the results of this study are very promising, it is still difficult to implement this in daily practice for selecting patients at risk for an ACL rupture. Although there is an increasing rate of knee MRI’s for all kinds of patients, it is still not routinely done. More and more professional sport clubs (such as football clubs) are having MRI’s of the knee taken as a routine sports checkup or pre-contract workup, which in the future could be used for pre-selecting patients at risk for an ACL rupture.

We understand that not every clinician will have time or the inclination to analyse the measurements we provide for all patients visiting the clinics. Therefore we would suggest that clinician could focus on the notch width index for selecting patients who are at greater risk for an ACL rupture, because this is a reproducible, simple measurement, every clinician is able to perform on a MRI of the knee. We found that when selecting a cut off point for the NWI of 26, 70 percent of the patients were patients with an ACL rupture, and only 30 percent of the patients had an intact ACL.

In addition, growing numbers of patients with an ACL rupture will have an MRI after the occurrence. The results of our study could therefore have important implications for the treatment and prevention of re-ruptures, which cause major health and economic issues [[4](#_ENREF_4)].

The role of the intercondylar notch in ACL injury was recently studied in children, concluding that a narrow notch with low NWI may be a risk factor for ACL tears in children suggesting that anterior impingement could indicate the need for notch-plasty to decrease the risk of recurrence following primary ACL reconstruction[[11](#_ENREF_11)]. Further research could focus on investigating if notch-plasty could decrease the re-rupture risk in adults.

In summary, this study showed that patients with a smaller intercondylar notch volume, smaller PCL volumes, a smaller bicondylar width and a smaller notch width index are more prone to sustaining an ACL rupture, when compared to a large group of control patients, who had knee injury during sports, but did not tear their ACL. Further research should investigate in how to use these results for selecting patients at risk for an ACL rupture and if these results have any implication for lowering the re-rupture risk.

**References**

1. Echografie en MRI. [web page]. 2011; https://[www.rivm.nl/medische-stralingstoepassingen/trends-en-stand-van-zaken/diagnostiek/echografie-en-mri](http://www.rivm.nl/medische-stralingstoepassingen/trends-en-stand-van-zaken/diagnostiek/echografie-en-mri). Accessed 23-08-2019, 2019.

2. Al-Saeed O, Brown M, Athyal R, Sheikh M (2013) Association of femoral intercondylar notch morphology, width index and the risk of anterior cruciate ligament injury. Knee Surg Sports Traumatol Arthrosc 21:678-682

3. Arendt E, Dick R (1995) Knee injury patterns among men and women in collegiate basketball and soccer. NCAA data and review of literature. Am J Sports Med 23:694-701

4. Barie A, Ehmann Y, Jaber A, Huber J, Streich NA (2019) Revision ACL reconstruction using quadriceps or hamstring autografts leads to similar results after 4 years: good objective stability but low rate of return to pre-injury sport level. Knee Surg Sports Traumatol Arthrosc;10.1007/s00167-019-05444-z

5. Barry KP, Mesgarzadeh M, Triolo J, Moyer R, Tehranzadeh J, Bonakdarpour A (1996) Accuracy of MRI patterns in evaluating anterior cruciate ligament tears. Skeletal Radiol 25:365-370

6. Bouras T, Fennema P, Burke S, Bosman H (2018) Stenotic intercondylar notch type is correlated with anterior cruciate ligament injury in female patients using magnetic resonance imaging. Knee Surg Sports Traumatol Arthrosc 26:1252-1257

7. Charlton WP, St John TA, Ciccotti MG, Harrison N, Schweitzer M (2002) Differences in femoral notch anatomy between men and women: a magnetic resonance imaging study. Am J Sports Med 30:329-333

8. Davis TJ, Shelbourne KD, Klootwyk TE (1999) Correlation of the intercondylar notch width of the femur to the width of the anterior and posterior cruciate ligaments. Knee Surg Sports Traumatol Arthrosc 7:209-214

9. Dienst M, Schneider G, Altmeyer K, Voelkering K, Georg T, Kramann B, et al. (2007) Correlation of intercondylar notch cross sections to the ACL size: a high resolution MR tomographic in vivo analysis. Arch Orthop Trauma Surg 127:253-260

10. Eggerding V, van Kuijk KS, van Meer BL, Bierma-Zeinstra SM, van Arkel ER, Reijman M, et al. (2014) Knee shape might predict clinical outcome after an anterior cruciate ligament rupture. Bone Joint J 96-B:737-742

11. Freychet B, Lakhal W, Daggett M, Bonnard C (2016) Intercondylar notch dysplasia in open-physis anterior cruciate ligament injuries: A case-control study. Orthop Traumatol Surg Res 102:203-206

12. Fung DT, Zhang LQ (2003) Modeling of ACL impingement against the intercondylar notch. Clin Biomech (Bristol, Avon) 18:933-941

13. Gwinn DE, Wilckens JH, McDevitt ER, Ross G, Kao TC (2000) The relative incidence of anterior cruciate ligament injury in men and women at the United States Naval Academy. Am J Sports Med 28:98-102

14. Hernigou P, Garabedian JM (2002) Intercondylar notch width and the risk for anterior cruciate ligament rupture in the osteoarthritic knee: evaluation by plain radiography and CT scan. Knee 9:313-316

15. Hewett TE, Myer GD, Ford KR (2006) Anterior cruciate ligament injuries in female athletes: Part 1, mechanisms and risk factors. Am J Sports Med 34:299-311

16. Hoteya K, Kato Y, Motojima S, Ingham SJ, Horaguchi T, Saito A, et al. (2011) Association between intercondylar notch narrowing and bilateral anterior cruciate ligament injuries in athletes. Arch Orthop Trauma Surg 131:371-376

17. Ireland ML, Ballantyne BT, Little K, McClay IS (2001) A radiographic analysis of the relationship between the size and shape of the intercondylar notch and anterior cruciate ligament injury. Knee Surg Sports Traumatol Arthrosc 9:200-205

18. Kaynak M, Nijman F, van Meurs J, Reijman M, Meuffels DE (2017) Genetic Variants and Anterior Cruciate Ligament Rupture: A Systematic Review. Sports Med 47:1637-1650

19. Li K, Du J, Huang LX, Ni L, Liu T, Yang HL (2017) The diagnostic accuracy of magnetic resonance imaging for anterior cruciate ligament injury in comparison to arthroscopy: a meta-analysis. Sci Rep 7:7583

20. Lindenfeld TN, Schmitt DJ, Hendy MP, Mangine RE, Noyes FR (1994) Incidence of injury in indoor soccer. Am J Sports Med 22:364-371

21. Myklebust G, Maehlum S, Holm I, Bahr R (1998) A prospective cohort study of anterior cruciate ligament injuries in elite Norwegian team handball. Scand J Med Sci Sports 8:149-153

22. Oshima T, Putnis S, Grasso S, Parker DA (2020) The space available for the anterior cruciate ligament in the intercondylar notch is less in patients with ACL injury. Knee Surg Sports Traumatol Arthrosc;10.1007/s00167-020-05921-w

23. Posthumus M, Collins M, September AV, Schwellnus MP (2011) The intrinsic risk factors for ACL ruptures: an evidence-based review. Phys Sportsmed 39:62-73

24. Shelbourne KD, Davis TJ, Klootwyk TE (1998) The relationship between intercondylar notch width of the femur and the incidence of anterior cruciate ligament tears. A prospective study. Am J Sports Med 26:402-408

25. Shen L, Jin ZG, Dong QR, Li LB (2018) Anatomical Risk Factors of Anterior Cruciate Ligament Injury. Chin Med J (Engl) 131:2960-2967

26. Simon RA, Everhart JS, Nagaraja HN, Chaudhari AM (2010) A case-control study of anterior cruciate ligament volume, tibial plateau slopes and intercondylar notch dimensions in ACL-injured knees. J Biomech 43:1702-1707

27. Staeubli HU, Adam O, Becker W, Burgkart R (1999) Anterior cruciate ligament and intercondylar notch in the coronal oblique plane: anatomy complemented by magnetic resonance imaging in cruciate ligament-intact knees. Arthroscopy 15:349-359

28. Stevenson H, Webster J, Johnson R, Beynnon B (1998) Gender differences in knee injury epidemiology among competitive alpine ski racers. Iowa Orthop J 18:64-66

29. Stijak L, Radonjic V, Nikolic V, Blagojevic Z, Aksic M, Filipovic B (2009) Correlation between the morphometric parameters of the anterior cruciate ligament and the intercondylar width: gender and age differences. Knee Surg Sports Traumatol Arthrosc 17:812-817

30. Sturnick DR, Vacek PM, DeSarno MJ, Gardner-Morse MG, Tourville TW, Slauterbeck JR, et al. (2015) Combined anatomic factors predicting risk of anterior cruciate ligament injury for males and females. Am J Sports Med 43:839-847

31. Sutton KM, Bullock JM (2013) Anterior cruciate ligament rupture: differences between males and females. J Am Acad Orthop Surg 21:41-50

32. van Eck CF, Kopf S, van Dijk CN, Fu FH, Tashman S (2011) Comparison of 3-dimensional notch volume between subjects with and subjects without anterior cruciate ligament rupture. Arthroscopy 27:1235-1241

33. van Eck CF, Martins CA, Vyas SM, Celentano U, van Dijk CN, Fu FH (2010) Femoral intercondylar notch shape and dimensions in ACL-injured patients. Knee Surg Sports Traumatol Arthrosc 18:1257-1262

34. van Kuijk KSR, Eggerding V, Reijman M, van Meer BL, Bierma-Zeinstra SMA, van Arkel E, et al. (2021) Differences in Knee Shape between ACL Injured and Non-Injured: A Matched Case-Control Study of 168 Patients. J Clin Med 10:

35. Whitney DC, Sturnick DR, Vacek PM, DeSarno MJ, Gardner-Morse M, Tourville TW, et al. (2014) Relationship Between the Risk of Suffering a First-Time Noncontact ACL Injury and Geometry of the Femoral Notch and ACL: A Prospective Cohort Study With a Nested Case-Control Analysis. Am J Sports Med 42:1796-1805

36. Wilson R, Barhorst AA (2018) Intercondylar Notch Impingement of the Anterior Cruciate Ligament: A Cadaveric In Vitro Study Using Robots. J Healthc Eng 2018:8698167

37. Wratten CJ, Tetsworth K, Hohmann E (2015) Three-Dimensional Femoral Notch Volume in Anterior Cruciate Ligament-Deficient Versus Anterior Cruciate Ligament-Intact Patients: A Matched Case-Control Study With Inter-gender Comparison. Arthroscopy 31:1117-1122

38. Zeng C, Gao SG, Wei J, Yang TB, Cheng L, Luo W, et al. (2013) The influence of the intercondylar notch dimensions on injury of the anterior cruciate ligament: a meta-analysis. Knee Surg Sports Traumatol Arthrosc 21:804-815

Figure legend:

Figure 1 Sagital view MRI of the knee, in which the PCL (left, green) and the ACL (right, blue) are outlined

Figure 2 Axial MRI image of the knee, A most proximal, B most distal of the intercondylar notch.

Figure 3 coronal MRI of the knee, with the described measurements. RL= Reference Line; NW = Notch Width; BW= Bicondylar width.
